# Bayesian Assessment of Corrosion-Related Failures in Steel Pipelines

**DOI:** 10.3390/e26121111

**Published:** 2024-12-19

**Authors:** Fabrizio Ruggeri, Enrico Cagno, Franco Caron, Mauro Mancini, Antonio Pievatolo

**Affiliations:** 1 Consiglio Nazionale delle Ricerche-Istituto di Matematica Applicata e Tecnologie Informatiche, I-20133 Milano, Italy; antonio.pievatolo@cnr.it; 2 Department of Management, Economics and Industrial Engineering, Politecnico di Milano, I-20156 Milano, Italy; enrico.cagno@polimi.it (E.C.); franco.caron@polimi.it (F.C.); mauro.mancini@polimi.it (M.M.)

**Keywords:** analytic hierarchy process, Poisson process, reliability of gas distribution networks

## Abstract

The probability of gas escapes from steel pipelines due to different types of corrosion is studied with real failure data from an urban gas distribution network. Both the design and maintenance of the network are considered, identifying and estimating (in a Bayesian framework) an elementary multinomial model in the first case, and a more sophisticated non-homogeneous Poisson process in the second case. Special attention is paid to the elicitation of the experts’ opinions. We conclude that the corrosion process behaves quite differently depending on the type of corrosion, and that, in most cases, cathodically protected pipes should be installed.

## 1. Introduction

This paper deals with the assessment of the probability of gas escape (hereinafter referred to as “failure”) from the steel pipelines in a gas distribution network, in order to support the estimation of its dependability and to the implementation of the maintenance and renewal policy. The problem of the failure analysis of pipelines, especially due to corrosion, has been the subject of several studies in the literature, especially in engineering. For example, ref. [1] considered corrosion in offshore and onshore oil and gas pipelines, and used a numerical analytical approach based on first-order reliability method and Monte Carlo simulation to assess uncertainty. Ref. [2] used numerical and analytical methods for limit state functions, with a particular interest in burst limit pressures, considering their relevance for oil and gas standards. The assessment of burst pressure standards for a pipeline was also considered in [3], using numerical analysis methods like finite elements and applying them to Algerian data. Our approach differs from those, since we are adopting a statistical approach based on stochastic models. Probabilistic methods, including those based on the Bayesian approach, have been used in the field. Indeed, stochastic modeling of consequences of corrosion of materials is an important aspect of structural reliability analysis, which has attracted a significant interest, especially in engineering. There are works on corrosion in reinforced concrete components, where our approach might be adapted and applied. Ref. [4] studied the reliability over the design life of a reinforced concrete beam, while ref. [5] was interested in corrosion-induced cracks, and applied advanced Bayesian simulation techniques, based on Hamiltonian Markov chain Monte Carlo methods. The importance of those studies is evident in [6], where the authors addressed the issue of the behavior of the reinforced concrete when subject to seismic events.

Furthermore, the corrosion process is a particular example of degradation, another field of reliability with rich literature, including books like [7] and applications like the one in [8] about the wear of cylinder liners in ships.

We are considering a Bayesian approach to the problem, since we are able to combine actual data with experts’ opinions. The Bayesian approach is widely used in reliability, with early works including the very influential book [9], followed by many others like [10], which strongly differs from the former not only because of the new areas of application but also because of the simulation techniques, mostly known as Markov Chain Monte Carlo, which were developed in the last 35 years.

In the paper a case study of a urban low pressure network laid in a metropolis of 1.5 million citizens is presented. Steel pipelines have very strong mechanical properties (failures are very rare), but they are easy prey for corrosive agents unless they are correctly protected.

After a brief description of the technical problem in Section 2, a Bayesian analysis based only on the observed number of failures during the period 1978–1997 is presented in Section 3. There, we focus on the probability that a failure occurs in a certain location, and that it is due to one among various types of corrosion processes. This information can be used as a quick reference to decide the kind of protection needed by a pipe for design purposes.

A more detailed analysis is carried out in Section 4 and Section 5, where we relate the corrosion process to the occurrence of failures, by taking into account both the times of occurrence of failures and the evolution of the network over time. The result is the possibility of following the temporal evolution of the reliability of a portion of network subject to a given corrosive agent for maintenance purposes.

In all cases, information provided by the experts is incorporated into the models according to the Bayesian paradigm.

## 2. Factors Related to the Probability of Failure

In the case under examination, the number of failures of the steel pipelines due to corrosion is very small (33 failures in the period 1978–1997 over a network of 275 km as of 1997). The scarceness of historical data has led us to use a methodology that takes into account all possible kinds of information. The principal source of information is the experts’ knowledge (grown inside the company that manages the gas distribution network), and the Bayesian framework [11] offers the tools to combine the historical data with the corporate memory.

From the available historical data inside the company and the interviews with experts, three principal elements that may be related to the failures of steel pipelines have been identified: the age of the pipe, the type of corrosion that led to the rupture of pipe, and the lay location of the pipe.

### 2.1. Age of the Pipe

Since corrosion is a process that develops progressively, time is an important factor of the corrosive phenomenon. Steel pipelines are subject to an electrolytic process that tends to reduce in thickness the walls of the pipe. Then, the pipe weakens and breaks because of the loss of its mechanical properties, with the consequent and extremely dangerous gas leak. Therefore, the age of the pipe, which can be correctly approximated by the lay date, is very important, because it represents the period of time the pipe has been exposed to this electrolytic phenomenon.

### 2.2. Type of Corrosion

Three types of corrosion have been determined: natural, galvanic, and by interference. Each of them is characterized by specific causes, and develops in fully different ways. This relates to their different relationships with the failure of the pipe.

#### 2.2.1. Natural Corrosion (*N*)

It is principally caused by the aggressive properties of the ground. For example, very wet ground is a good conductor and, as such, facilitates the development of the electrolytic phenomenon on the steel.

#### 2.2.2. Galvanic Corrosion (*G*)

A gas distribution network is very inhomogeneous because it is made up of very different materials (treated cast iron, spheroid graphite, traditional cast iron, polyethylene and so on). When a steel pipe is connected to a cast iron pipe, if the insulating material, that constitutes the principal protection of the pipe, is not perfectly efficient, a galvanic corrosion caused by the contact of the two materials can take place. In particular, there is a potential difference between cast iron and steel, where the latter assumes the anodic behavior and corrodes, while the former assumes the cathodic one.

#### 2.2.3. Corrosion by Interference (*I*)

This is also called corrosion by stray current, because it is due to the presence of stray currents in the ground coming from other badly isolated electrical plants (for example, streetcar substations or train stations). These currents discharge themselves on the steel pipe, increasing the corrosion rate by various orders of magnitude. In other cities, other corrosive agents also hidden in the ground can be very important (especially near the sea), but not in the present case.

### 2.3. Lay Location

The propensity to a particular type of corrosion is not the same all over the city, but changes according to the place where the pipe is laid. In fact, in some places, stray currents in the ground are significantly higher than in other places, which increases the propensity to a failure due to corrosion by interference. Therefore, two different areas can be determined: zone A, characterized by the presence of streetcar substations or rail stations, and zone B, without them.

## 3. Bayesian Inference on the Probability of Failure by Zone and by Type of Corrosion

### 3.1. Comparing the Observed Number of Failures by Zone and by Type of Corrosion

The geometry of the failures across the city is not uniform. In Section 2.3, we have argued that the probability of a failure is higher where stray currents are present, which especially happens near streetcar substations, rail stations, and so on.

#### 3.1.1. Closeness of Streetcar Substations

Near streetcar substations, the trunk of negative electric cables used to feed the streetcar line is hidden underground. The streetcar-feeding current is generated in the substation, goes through the aerial line (positive cables), and is transformed into power by the streetcar; then, it goes through the steel streetcar tracks (negative cables) to close the circuit in the substation.

The circuit is actually closed, in the last hundred meters, by underground cables connected to the steel streetcar tracks and to the generator of the substation. These cables generate stray currents because of the bad isolation.

#### 3.1.2. Closeness of Rail Stations and Lines

In the case of rail stations, the stray currents derive not only by the bad isolation of the tracks, but also by the strong electric field coming from the passage of the train. Therefore, the train traffic is proportional to the mean intensity of the stray currents.

In conclusion, we determined two areas: zone A, where there is a trunk of negative electric cables used to feed the streetcar line or near rail stations or rails, and zone B, which is all other areas.

In order to compare the number of failures by zone, the number of observed failures must be divided by the lengths of the gas distribution networks in zone A and in zone B, respectively. Since this datum is unavailable, we approximate it by the area (in squared kilometers) of the two zones.

The observed number of failures by zone and by type of corrosion per kilometer and per year (named here “failure rate”) is summarized in Table 1.

It is reasonable to expect that the “failure rate” by natural corrosion in zone A is comparable to that in zone B, but this is not the case for the observed failures, probably because of incorrect classifications made by the repairing teams. This will not affect the estimate of the marginal probability that a failure occurs in zone A, whereas it will bias the estimates of the marginal probabilities that a corrosion occurs of a given type, as well as the estimates of some conditional probabilities. Therefore, some care must be taken in interpreting the result, even if the misclassification bias will be partially counterbalanced by the prior opinion of the experts.

### 3.2. Prior Probability Assessment: The Analytic Hierarchy Process

Experts’ prior estimates of the zone probability (from now on, we will use the shorthand “zone probability” to stand for “probability that a failure occurs in a given zone”) have been achieved by means of the pairwise comparison technique of the Analytic Hierarchy Process (AHP) [12,13]. AHP is, at the same time, a useful and widely used methodology, and a controversial one, as recognized by many authors, including the proposer [14]. Ref. [15] presented a non-exhaustive list of 150 papers using AHP up to that time, illustrating critical and positive aspects of the methodology.

On the positive side, AHP helps in specifying prior probabilities when the experts are able to provide only qualitative judgments and not quantitative ones, like mean and variance of a prior distribution. Given a set of *n* events, the experts are asked to compare them pairwise, making linguistic statements about the likelihood of occurrence of one with respect to the other. Each judgment is transformed into a number, and a square matrix of order *n* is obtained with all the comparisons. The eigenvector of dimension *n*, corresponding to the largest eigenvalue of the matrix, is then normalized so that its elements sum to 1, and they represent the probabilities of occurrence of each of the *n* events. Mathematical details can be found in the books mentioned earlier.

As anticipated, there are some controversial aspects about AHP. First, of all, the scale of linguistic judgments has to be clearly specified, especially regarding the increased order of the possible sentences. For example, all of the experts should understand that “much more likely” means that it is less likely than “clearly more likely”. Training with simple examples is strongly suggested so that all of the experts agree on the scale and each statement, like that “clearly more likely” has more-or-less the same meaning for all of them. The assignment of numbers to statements is arbitrary, from their range to the value assigned to each judgment. The matrix could also have some inconsistencies, like values $a_{ij}$ and $a_{jk}$ assigned to comparisons between events *i* and *j* and between *j* and *k*, respectively, but $a_{ik}\neq a_{ij}a_{jk}$ when comparing events *i* and *k*. Measures of inconsistency have been proposed, as well as methods to attenuate the problem. Ref. [16] considered propensity to gas escape in cast iron pipelines laid under different conditions, like diameter, depth, and location. They used AHP to obtain opinions from the experts about the propensity for each condition (eight, given that each feature was taking two possible values). They considered a homogeneous Poisson process for the failure times, since cast iron is not subject to corrosion, as steel pipes, and propensity to fail can be assumed constant over time. Following the Bayesian approach, gamma priors were considered for the parameters of the different conditions and their parameters were chosen so that their mean and variance were given by the sample mean and variance of the opinions of the experts. Those priors were then validated by the company’s experts. We are taking a similar approach here.

The team of experts was formed by two technicians and two engineers: the technicians assess the conditions of the pipes after an excavation and decide what action to take; the engineers have an overall knowledge of the technical and information management problems that arise inside the company.

Two types of assessments were requested: the probability of a failure in the two zones, and the probability of a type of corrosion conditioned to the zone. All questions were phrased in terms of relative probabilities. For example, the first one was: “In your opinion is a failure more likely to happen in zone A or in zone B? How much more likely?”.

In order to fully exploit the answers, a linguistic judgment scale has been referred to the numerical scale {1,3,5,7,9}, and the judgments have been used to obtain the prior probability assessment. The five degrees of the linguistic judgment scale were: “equally likely”, “a little more likely”, “much more likely”, “clearly more likely”, “definitely more likely”.

The experts answered considering the number of failures observed and that the areas near streetcar substations or rail stations are under close control. In spite of this, the subjective probability of failure in zone A was assessed as about four times greater than that in zone B.

The second part of the questionnaire regarded the comparison among the three types of corrosion by zone. The typical question was: “In an area with (without) streetcar substations or rail stations is it more likely to have natural or galvanic corrosion? How much more likely?”.

From the questionnaire, P(N|A), P(G|A), P(I|A), P(N|B), P(G|B), and P(I|B) were obtained, while the zone probabilities conditioned to the type of corrosion were derived as follows:

$$\begin{array}{ccc} P(C) & = & P(C|A)P(A)+P(C|B)P(B)\\ P(A|C) & = & \frac{P(C|A)P(A)}{P(C)} \end{array}$$

where C∈{I,N,G} and the indicated probabilities have an obvious meaning.

The prior estimates and their standard deviations are shown in Table 2.

### 3.3. Posterior Probabilities Distinguishing the Zone

So far, from the expert judgments, it has emerged that the propensity to failure due to different types of corrosion is not the same for the two zones (in particular for the corrosion by interference). In the next two paragraphs, we derive the posterior zone probability, both marginal and conditioned to the type of corrosion.

#### 3.3.1. Zone Probability

Let us denote by *p* the probability that a failure occurs in zone A, so P(A)=p. Denoting by $n_{A}$ the number of failures in zone A, and conditioning upon the total number of failures *n*, the phenomenon is described by a binomial distribution with *n* trials and parameter *p*,

(1) p(nA|n,p)=nnApnA(1−p)n−nA

The Beta distribution is conjugate to the binomial, so we take p∼Beta(a,b) a priori, where *a* and *b* are chosen consistently with the prior assessments in Table 2. Then, $p∼\mathrm{Beta}(a^{\prime },b^{\prime })$ a posteriori, with $a^{\prime }=a+n_{A}$ and $b^{\prime }=b+n-n_{A}$.

The expected loss, under quadratic loss function, is minimized by the posterior mean of *p*,

$$E\left(p|n,n_{A}\right)=a^{\prime }/\left(a^{\prime }+b^{\prime }\right)=27.460/\left(27.460+29.866\right)=0.4790.$$

The results are compared in Table 3.

The strong influence of the streetcar substations and rail stations on the probability of failure of the steel pipelines is confirmed a posteriori. In fact, on average, around 48% of the failures is concentrated on 12% of the city area. The small difference between posterior means and MLEs gives us confidence that the failures have been classified correctly by zone.

#### 3.3.2. The Zone Probability Conditioned to the Type of Corrosion

Since the way to obtain the zone probability conditioned to the type of corrosion is the same as the previous paragraph, here, we report only the results in Table 4.

### 3.4. Type of Corrosion: Marginal Posterior Probabilities

The progress of the electrolytic phenomenon depends strictly on the type of corrosion, thus it is necessary to study which type of corrosion is more frequent. A corrosion of a given type occurs according to the probabilities $p_{I}\equiv P\left(I\right)$, $p_{G}\equiv P\left(G\right)$, and $p_{N}\equiv P\left(N\right)$.

As in Section 3.3.1, conditional upon *n*, the number of failures by type of corrosion $\left(n_{I},n_{G},n_{N}\right)$ has a multinomial distribution with parameters $p_{I}$, $p_{G}$, and $p_{N}=1-p_{I}-p_{G}$. Then, we have

p(nN,nG|n,pN,pG)=nnNnGpNnNpGnG(1−pN−pG)n−nN−nG

The generalization of the Bayesian procedure followed in Section 3.3.1 is obtained by giving a Dirichlet prior distribution to $\left(p_{N},p_{G}\right)$ (which becomes a Beta distribution when there are only two classes),

$$p\left(p_{N},p_{G};a_{N},a_{G},a_{I}\right)=\frac{\Gamma (a_{\mathrm{tot}})}{\Gamma \left(a_{N}\right)\Gamma \left(a_{G}\right)\Gamma \left(a_{I}\right)}p_{N}^{a_{N}-1}p_{G}^{a_{G}-1}{\left(1-p_{N}-p_{G}\right)}^{a_{I}-1}$$

where $a_{\mathrm{tot}}=a_{N}+a_{G}+a_{I}$, and where the hyperparameters are determined by matching the prior mean and variances in Table 2 to those given by the model as *c* varies within {I,G,N},

$$\begin{array}{ccc} E(p_{c}) & = & \frac{a_{c}}{a_{\mathrm{tot}}}\\ \mathrm{Var}(p_{c}) & = & \frac{a_{c}\left(a_{\mathrm{tot}}-a_{c}\right)}{a_{\mathrm{tot}}^{2}\left(a_{\mathrm{tot}}+1\right)} \end{array}$$

The posterior distribution of $\left(p_{N},p_{G}\right)$ is again Dirichlet with parameters

$$\begin{array}{ccc} a_{c}^{\prime } & = & a_{c}+n_{c}c\in \left\{I,G,N\right\}\\ a_{\mathrm{tot}}^{\prime } & = & a_{\mathrm{tot}}+n \end{array}$$

The comparison of the various means is reported in Table 5.

Here, the posterior means differ from the MLE’s much more markedly than those in Table 3, showing that the experts’ opinions do not agree with the classification of the failures by type of corrosion, which reinforces our suspects of misclassifications.

## 4. A Model for the Occurrence of Failures

### 4.1. Available Data and Notation

The introduction of the age of the pipes in the analysis requires considering additional data: the times of occurrence of the failures, the installation dates, and the lengths of subnetworks laid in different periods. Because the network existing in 1978–1997 (the period for which the records are reliable) is also composed of subnetworks laid very early, the periodic growth of the network since its outset must be considered. A yearly record of the increments in kilometers of the network is available from the gas company, regardless of the zone, because no systematic attempt to distinguish zone A from zone B was ever made by the company.

We set out the mathematical notation at this point, in order not to interrupt the flow of ideas in the following. Since we are examining a corrosion process, it makes sense to partition the networks into subnetworks whose components have been laid in the same period (e.g., in the same year). Suppose that there are *r* subnetworks. Denote these subnetworks by their installation year $\left\{s_{i},i=1,\ldots ,r\right\}$ and their lengths (in kilometers) by $l_{i}$. When we refer to a generic subnetwork, we index it by *s* only without subscript, and we denote its length by $l_{s}$.

The time of the *k*-th observed failure in subnetwork $s_{i}$ is denoted by $t_{ick}$, where c∈{I,G,N} indicates the type of corrosion that caused the failure: by interference, galvanic, or natural. The index *k* ranges from 1 to $n_{ic}$.

The observation interval for the subnetwork is denoted by $\left[\max\left\{T_{0},s_{i}\right\},T_{1}\right]$, where $T_{0}$ is 1 January 1978 and $T_{1}$ is 31 December 1997.

### 4.2. Modeling a Single Failure

We build a stochastic model for the observed failures in the whole network, starting from the modeling of the time of occurrence of a single failure. A failure in a subnetwork *s* occurs as the final result of a sequence of events: first, some external intervention causes a damage to the protection of a pipe after a random time *u* since the installation of the pipe; second, because of the damage, a corrosion process of type *c*, according to the location, takes place until the failure of the pipe after a random time *v*, independently of *u*. The actual time of failure can then be written as t=s+u+v, so that the probability density of *t* is the convolution of the probability densities of *u* and of *v*. Denote these densities by $h(\cdot |\lambda ,l_{s})$ and $g(\cdot |M_{c},\beta_{c})$, respectively, where the subscript *c* indicates the dependence of the distribution of the corrosion time *v* on the type of corrosion that has taken place. The probability density of *t* is then

(2) $$\begin{array}{ccc} f(t|\lambda ,M_{c},\beta_{c},l_{s},s) & = & \int_{0}^{t-s}g\left(t-s-u|M_{c},\beta_{c}\right)h\left(u|\lambda ,l_{s}\right)du\\ & = & \int_{s}^{t}g\left(t-z|M_{c},\beta_{c}\right)h\left(z-s|\lambda ,l_{s}\right)dz \end{array}$$

where we have set z=s+u.

We chose $h(\cdot |\lambda ,l_{s})$ to be negative exponential, because the absence of damages up to a given time does not change the probability of occurrence, i.e., the hazard function is constant. Then,

$$h\left(u|\lambda ,l_{s}\right)=l_{s}\lambda e^{-l_{s}\lambda u},$$

where the presence of the factor $l_{s}$ implies that the mean time to damage is shorter for longer subnetworks. The density of *v* must belong to a family of densities that allow for increasing hazard functions, because of the presence of the corrosion process. The Weibull family is widely used in this kind of situation, so we set

$$g\left(v|M_{c},\beta_{c}\right)=M_{c}\beta_{c}v^{\beta_{c}-1}e^{-M_{c}v^{\beta_{c}}}.$$

The integral in (2) cannot be solved explicitly, so we will use numerical integration when $f(\cdot |\lambda ,M_{c},\beta_{c},l_{s},s)$ is needed.

### 4.3. Combining the Single Failure Models

When corrosion has led to the rupture of a pipeline section having a certain lay date $s_{i}$, it is replaced by a new section. Therefore, subnetwork $s_{i}$ becomes shorter at every rupture event until all of its parts are substituted, hence its life span is finite. During its life, a random number $N_{ic}$ of failures due to corrosion of type *c* occur. Conditional upon $N_{ic}$, we can regard the failure times as independent random variables, all with density (2), because the subnetworks are large and the failures occur at different locations. In our case, $N_{ic}$ is unknown, and we have observed the failures only in the interval $\left[\max\left\{T_{0},s_{i}\right\},T_{1}\right]$, so the joint density function of $t_{ic}=\left\{t_{ick},k=1,\ldots ,n_{ic}\right\}$ and $n_{ic}$ must be found by averaging the conditional density over $N_{ic}$, which takes the following form, as shown in Appendix A:

(3) $$\begin{array}{ccc} f(t_{ic},n_{ic}|N_{ic},\lambda ,M_{c},\beta_{c},l_{i},s_{i}) & = & \prod_{k=1}^{n_{ic}}f\left(t_{ick}|\lambda ,M_{c},\beta_{c},l_{i},s_{i}\right)\\ & & \cdot {\left[1-D_{ic}\right]}^{N_{ic}-n_{ic}}\cdot \frac{N_{ic}!}{(N_{ic}-n_{ic})!} \end{array}$$

where

(4) $$D_{ic}=\int_{\max\{T_{0},s_{i}\}}^{T_{1}}f\left(t|\lambda ,M_{c},\beta_{c},l_{i},s_{i}\right)dt.$$

Suppose now that $N_{ic}$ is Poisson distributed with parameter $l_{i}\nu_{c}$, where $l_{i}$ accounts for the different lengths of the subnetworks, then the marginal joint density of $t_{ic}$ and $n_{ic}$ is

(5) $$f\left(t_{ic},n_{ic}|\lambda ,M_{c},\beta_{c},l_{i},s_{i}\right)=\prod_{k=1}^{n_{ic}}f\left(t_{ick}|\lambda ,M_{c},\beta_{c},l_{i},s_{i}\right)\cdot e^{-l_{i}\nu_{c}D_{ic}}{\left(l_{i}\nu_{c}\right)}^{n_{ic}}.$$

In a nutshell, Equation (5) is the probability density for what is observed in the window $\left[\max\left\{T_{0},s_{i}\right\},T_{1}\right]$ from a Poisson process on $\left[s_{i},+\infty \right)$ with intensity function given by $l_{i}\nu_{c}f\left(\cdot |\lambda ,M_{c},\beta_{c},l_{i},s_{i}\right)$. The fact that the intensity function decays to zero as time goes on is an approximation of the limited life span of the subnetwork $s_{i}$. Of course, the marginal distribution of $n_{ic}$ is Poisson with parameter $l_{i}\nu_{c}D_{ic}$. For the theory of Poisson processes and its applications see, e.g., [17], while [18] provide a thorough Bayesian analysis.

### 4.4. The Likelihood Function and the Bayesian Inference

The likelihood function for the unknown parameter vector θ=(λ,M,β,ν), where $M=(M_{I},M_{G}$, $M_{N})$, and a similar definition holds for β and ν, is obtained from (5) by taking the product over the indices *i* and *c*,

(6) $$\begin{array}{ccc} L(\theta ;t_{I},t_{G},t_{N}) & \propto & \lambda^{n_{\cdot \cdot }}e^{-\sum_{i}\sum_{c}l_{i}\nu_{c}D_{ic}}\prod_{c}\left\{{\left(M_{c}\beta_{c}\right)}^{n_{\cdot c}}\prod_{i=1}^{r}\right.\\ & & \left.\left[{\left(l_{i}^{2}\nu_{c}\right)}^{n_{ic}}\prod_{k=1}^{n_{ic}}\int_{s_{i}}^{t_{ick}}{\left(t_{ick}-z\right)}^{\beta_{c}-1}e^{-M_{c}{\left(t_{ick}-z\right)}^{\beta_{c}}}e^{-l_{i}\lambda \left(z-s_{i}\right)}dz\right]\right\} \end{array}$$

The vectors $t_{c}$, c∈{I,G,N}, appearing on the left-hand side of (6), contain the times of observed failures after a corrosion of type *c*; the dot subscripts indicate that the subscript in that position has been summed over; when $n_{ic}=0$, the product with index *k* in the second line is set to 1.

For the Bayesian inference, we assign prior distributions to all of the parameters in θ, and assume they are a priori independent, since they describe phenomena of a different nature. In particular, we take $\lambda ∼G(a^{\left(\lambda \right)},b^{\left(\lambda \right)})$. As *c* varies inside {I,G,N}, we put $M_{c}∼G\left(a_{c}^{\left(M\right)},b_{c}^{\left(M\right)}\right)$, $\beta_{c}∼G\left(a_{c}^{\left(\beta \right)},b_{c}^{\left(\beta \right)}\right)$, and $\nu_{c}∼G\left(a_{c}^{\left(\nu \right)},b_{c}^{\left(\nu \right)}\right)$, where the superscripts identify which parameter the hyperparameters are to be referred to.

### 4.5. The Probabilities of Zone and of Type of Corrosion

A formal connection between this section and Section 3 can be seen immediately. Let us split $\nu_{c}$ into the sum of two parameters, for example $\nu_{Ac}$ and $\nu_{Bc}$, c∈{G,I,N}, and perform the same with the observed values of $n_{ic}$, and with $l_{i}$ for all i=1,…,r, supposing for the moment that the lengths of the subnetworks laid in zones A and B are available. From Section 4.3, $n_{zic}$ conditional on θ is Poisson with mean $l_{zi}\nu_{zc}D_{zic}$, with the subscript *z* indicating the zone. Then, $n_{z\cdots }$ is Poisson with mean $\sum_{i}\sum_{c}l_{zi}\nu_{zc}D_{zic}$, so, conditioning to $n_{\cdots }\equiv n$, $n_{z\cdots }$ has a binomial distribution with number of trials $n_{\cdots }$ and probability of “success”

$$p=\frac{\sum_{i}\sum_{c}l_{zi}\nu_{zc}D_{zic}}{\sum_{i}\sum_{c}l_{Ai}\nu_{Ac}D_{Aic}+\sum_{i}\sum_{c}l_{Bi}\nu_{Bc}D_{Bic}}$$

where *p* is the same parameter that appears in Section 3.3.1. The same property, yielding a multinomial distribution as in Section 3.4, holds for $n_{\cdots c}$ conditional on $n_{\cdot \cdot \cdot }$.

## 5. Elicitation of the Experts’ Opinions and Results

Unlike the zone probability, the parameters λ, $M_{c}$, and $\beta_{c}$ (c∈{I,G,N}) are not easily related to an observable event whose probability can be assessed by an expert without difficulty.

As regards $M_{c}$ and $\beta_{c}$, following [19], the experts were asked to select an interval from a given list for the duration of the corrosion until failure (that is, an interval for *v* as defined in Section 4.2) and an attached degree of belief 100α% to be chosen among 95%, 85%, and 75%. From each interval, a pair $\left(M_{c},\beta_{c}\right)$ was found by assigning a value of (1−α)/2 to the tail probabilities. The averages over the experts gave the prior means and standard deviations of $M_{c}$ and $\beta_{c}$, as *c* varies within {I,G,N}, are presented in Table 6. The choice of the size of the intervals and the probabilities to be attached was dictated by the need to provide the experts, not acquainted with quantitative methods and prior elicitation, a time period sufficiently large to allow them to think about possible failures and three levels of probabilities ranging from a good chance of event (75%) to a great one (95%).

As for λ, where 1/λ is the mean time to damage of the protection of one kilometer of pipeline, the experts were asked to provide the probability that the protection of 5 km of pipeline is not damaged after 6, 15, 30, and 90 years. The value of λ for each expert was then found as the one that gave the best fit of exp(−5λy), where y∈{6,15,30,90}, to the elicited probabilities. The average of λ over the experts is 0.621 × 10^−2^, with a standard deviation of 0.182 × 10^−2^.

From the a priori means and variances, we can derive the hyperparameters of the Gamma prior densities, so we have $a^{\left(\lambda \right)}=11.6$ and $b^{\left(\lambda \right)}=1867$, $a_{I}^{\left(M\right)}=0.30$ and $b_{I}^{\left(M\right)}=14.5$, $a_{G}^{\left(M\right)}=1.28$ and $b_{G}^{\left(M\right)}=556$, $a_{N}^{\left(M\right)}=2.13$ and $b_{N}^{\left(M\right)}=6982$, $a_{I}^{\left(\beta \right)}=3.00$ and $b_{I}^{\left(\beta \right)}=0.86$, $a_{G}^{\left(\beta \right)}=8.63$ and $b_{G}^{\left(\beta \right)}=3.09$, and $a_{N}^{\left(\beta \right)}=14.04$ and $b_{N}^{\left(\beta \right)}=4.74$. The distributions of the $M_{c}$’s are concentrated on different parts of the real line, whereas the distributions of the $\beta_{c}$’s have approximately the same location, but are not equally dispersed. In particular, the experts seem to less clearly perceive the duration of the corrosion by interference.

The estimation of the posterior mean and standard deviation of the unknown parameters were carried out by Markov Chain Monte Carlo using the *mcmc* package [20], with numerical integration by quadrature for the computation of the intractable factors in the likelihood function (6), which are those involving $D_{ic}$ (see also Appendix A) and the kernel of the lifetime density (2). Since parameters $\nu_{c}$ are nuisance parameters, they were integrated out analytically in the actual calculations, which depend only on $a_{c}^{\left(\nu \right)}$ and $b_{c}^{\left(\nu \right)}$. We set both these parameters to 1 for all *c*, so that $E(\nu_{c})=1$; other values had very little influence on the results. The factors in the likelihood depending on parameters $\nu_{c}$ are replaced by $1/{\left(1+\sum_{i}l_{i}D_{ic}\right)}^{n_{\cdot c}+1}$.

The posterior means and standard deviations of the parameters appear in Table 7. The main change with respect to the prior summaries of Table 6 is that galvanic corrosion and corrosion by interference have swapped places, a result which we comment on at the end of this section. Posterior standard deviations mostly show a small reduction in uncertainty. A further reduction could be obtained using more specific corrosion models and improving the method for type classification by the operators. We also remark that the failure observation period is one third of the network-lifetime.

The advantage of using the lifetime distribution (2) can be appreciated when a maintenance decision must be made. Suppose that priority for substitution must be assigned to two parts of the network of equal length *l* laid in zone A and B, respectively, and let the lay year of the two parts be $s_{1}>s_{2}$. A decision based on the posterior zone probability derived in Section 3.3.1 would be to substitute the part in zone A irrespective of $s_{1}$ and $s_{2}$. Alternatively, one can consider the hazard functions associated with the mixture densities

$$\sum_{c}P\left(c|A\right)f\left(t|\lambda ,M_{c},\beta_{c},l,s_{1}\right)\;\mathrm{and}\;\sum_{c}P\left(c|B\right)f\left(t|\lambda ,M_{c},\beta_{c},l,s_{2}\right)$$

where P(c|A) and P(c|B) are the probabilities of corrosion conditioned to the zone derived in Section 3. These mixtures can be seen as an approximation to

$$\sum_{c}\frac{\nu_{Ac}}{\nu_{A\cdot }}f\left(t|\lambda ,M_{c},\beta_{c},l,s_{1}\right)\;\mathrm{and}\;\sum_{c}\frac{\nu_{Bc}}{\nu_{B\cdot }}f\left(t|\lambda ,M_{c},\beta_{c},l,s_{2}\right)$$

that is, the lifetime distributions of the time to failure of a part of network that fails exactly once. In this way we avoid estimating the parameters $\nu_{zc}$.

A posteriori estimates of the mixing coefficients P(c|z) are available from Section 3, whereas the pointwise estimate of the density functions is obtained by averaging the sequence $f(t|\lambda^{\left(i\right)},M_{c}^{\left(i\right)},\beta_{c}^{\left(i\right)},l,s)$ across MCMC iterations, indexed by *i*. The comparison of the hazard functions might result in the substitution of the part in zone B instead of that in zone A.

The typical reliability profile of the network might be represented by the hazard function associated with the mixture density

(7) $$g\left(t\right)=\sum_{c}P\left(c\right)f\left(t|\lambda ,M_{c},\beta_{c},l,0\right)$$

for some reference length *l*, where the unknown quantities are substituted by estimates as before, and again P(c) approximates $\nu_{\cdot c}/\nu_{\cdot \cdot }$.

**Figure 1:**
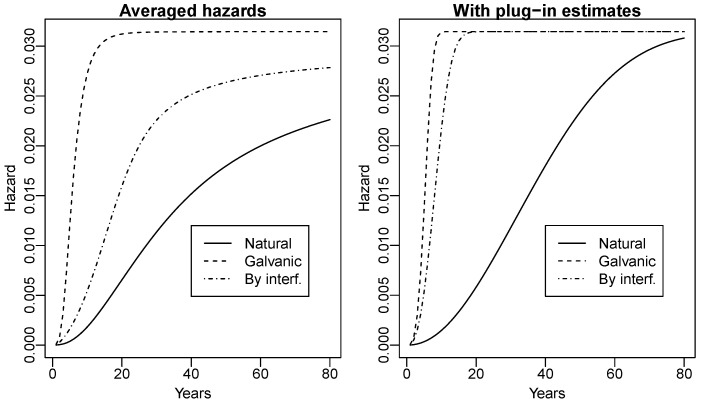
Hazard functions associated with $f(t|\lambda ,M_{c},\beta_{c}$, 5,0) for any c∈{N,G,I}. Left panel: posterior means of hazard functions for each failure type obtained from the MCMC simulation; right panel: computed by inserting the posterior means of parameters (plug-in estimate).

The plots of the hazard functions associated with $f(t|\lambda ,M_{c},\beta_{c},5,0)$ for any *c* appear in Figure 1. The difference between the two panels is mainly due to the asymmetry of the posterior distributions of the $M_{c}$ and $\beta_{c}$ parameters, which can be observed in the histograms of the MCMC simulations. Because of asymmetry, the sample averages of parameters used as plug-in estimators are larger than the medians and select hazard functions that increase more rapidly.

The left panel of Figure 1 highlights that the tails of the lifetime distributions are exponential, so that the hazard functions stay constant after some years; besides, the hazard function of the natural corrosion increases less fast than the other two, as expected. This tail behavior has a straightforward interpretation: if, after installing a 5 km pipeline, we do not see any failure for a certain period (whose length depends on the type of corrosion), we may assume that the protection has not been damaged, so the risk of a failure is equal to that of damage. Another feature is that the hazard function of the corrosion by interference is closer to that for galvanic corrosion than the hazard function of the natural corrosion, so that the data confirm the experts’ opinions (see beginning of Section 5). The less steep slope of the hazard function for the corrosion by interference with respect to the galvanic one reflects a longer mean time to failure; again, this might be imputed to failures by natural corrosion that happened in zone A and that were incorrectly classified as due to interference.

## 6. Concluding Remarks

A methodology for the assessment of the probability of gas escape from steel pipelines has been presented to support the implementation of the maintenance and renewal policy of a urban gas distribution network. We identified the factors that influence reliability, and focused first on the type of corrosion and the lay location (for design purposes), and then also included the age of the pipe in the analysis (for maintenance purposes).

The developed methodology answered the requirement to combine, according to the Bayesian paradigm, scarce information derived from historical data with the experts’ knowledge grown inside the company during the last running periods.

At the design stage, a simple beta-binomial model proved sufficient, showing that streetcar substations greatly increase the probability of a fast corrosion; therefore, considering that it is not easy to tell whether a corrosion by interference can take place in a given location, newly installed pipelines should be cathodically protected.

At a maintenance stage, because of the presence of corrosion, we estimated a non-homogeneous Poisson process that accounts for the age of the pipes. The type of corrosion proved influential on the shape of the intensity function of the process, so that it is reasonable to compare the hazard functions derived from it when a priority decision for substitution must be made in presence of information on the most likely type of corrosion. The computation required for the estimation of the parameters are intensive in this case, but perfectly feasible thanks to modern computer power.

The real case study presented emphasized the relevance of the practical solution, to be reused in other industrial cases.

## Figures and Tables

**Table 1:** Failure rate by zone and by type of corrosion.

	Natural (*N*)	Galvanic (*G*)	By Interference (*I*)
Zone A (12 km^2^)	0.583	0.083	0.500
Zone B (88 km^2^)	0.068	0.057	0.091

**Table 2:** Subjective assessments of zone probabilities and type of corrosion.

	Mean	Std. Dev.
P(A)	0.7938	0.1962
P(B)	0.2063	0.1962
P(A|N)	0.6133	0.2114
P(A|G)	0.6221	0.2168
P(A|I)	0.9581	0.0574
P(N)	0.1636	0.0403
P(G)	0.2767	0.1298

**Table 3:** Comparison of historical, prior and posterior mean of *p*.

		Historical (MLE)	Prior	Posterior
*p*	(zone A, 12 km^2^)	0.4528	0.7938	0.4790
1−p	(zone B, 88 km^2^)	0.5472	0.2062	0.5210

**Table 4:** Comparison of historical, prior and posterior mean of *p* conditioned to the type of corrosion.

	Historical (MLE)	Prior Mean	Prior Std. Dev.	Posterior Mean	Posterior Std. Dev.
P(A|N)	0.5385	0.6133	0.2114	0.5662	0.1065
P(A|G)	0.1667	0.6221	0.2168	0.4125	0.0351
P(A|I)	0.4286	0.9581	0.0574	0.6700	0.0909

**Table 5:** Comparison of the historical, prior and posterior mean of $p_{c}$, c∈{I,G,N}.

	Historical (MLE)	Prior Mean	Prior Std. Dev.	Posterior Mean	Posterior Std. Dev.
P(N)	0.3940	0.1636	0.0403	0.2290	0.0388
P(G)	0.1818	0.2767	0.1298	0.2498	0.0400
P(I)	0.4242	0.5597	0.1565	0.5212	0.0461

**Table 6:** Prior assessment of $M_{c}$ and $\beta_{c}$, c∈{I,G,N}.

Type of Corrosion	Parameter	Prior Mean	Prior Std. Dev.
Natural	*M*	0.305 × 10^−3^	0.209 × 10^−3^
	β	2.96	0.79
Galvanic	*M*	0.229 × 10^−2^	0.203 × 10^−2^
	β	2.79	0.95
By interference	*M*	0.210 × 10^−1^	0.381 × 10^−1^
	β	3.48	2.01

**Table 7:** Posterior means and standard deviations of λ, $M_{c}$, and $\beta_{c}$, c∈{I,G,N}.

Type of Corrosion	Parameter	Posterior Mean	Posterior Std. Dev.
All	λ	0.619 × 10^−2^	0.181 × 10^−2^
Natural	*M*	0.338 × 10^−3^	0.202 × 10^−3^
	β	2.21	0.42
Galvanic	*M*	0.235 × 10^−2^	0.163 × 10^−2^
	β	3.44	0.70
By interference	*M*	0.266 × 10^−2^	0.417 × 10^−2^
	β	2.68	1.19

## Data Availability

Restrictions apply to the availability of these data.

## Appendix A. Derivation of Expressions

We derive (3), and other expressions, by omitting all quantities and notation that are unnecessary for this purpose. Conditional on *N*, (3) represents the probability density of *n* failures at $t_{1},\ldots ,t_{n}$ inside $\left(\max\left\{T_{0},s\right\},T_{1}\right)$, and of the remaining N−n failures outside. This occurs if *n* failures are first assigned to the interval with binomial probability with success probability *D* and *N* trials, where *D* is the probability that a failure falls inside $\left(\max\left\{T_{0},s\right\},T_{1}\right)$, given by (4). Then, given *n*, the joint density of the failures is that of the order statistics from f(t) restricted to the interval. Taking the product of these two factors, we have

NnDn(1−D)N−n×n!∏k=1nf(tk)D

which coincides with (3) after simplifications.

We now consider *D* at (4). After writing f(t|λ,M,β,l,s) explicitly, we can interchange the order of integration and obtain the following expression:

$$\int_{s}^{T_{1}}\left[\int_{\max\{z,T_{0}\}}^{T_{1}}M\beta {\left(t-z\right)}^{\beta -1}e^{-M{\left(t-z\right)}^{\beta }}dt\right]l\lambda e^{-l\lambda (z-s)}dz$$

where the inside integral can be solved explicitly, since the integrand is a Weibull density, so we have

$$\int_{s}^{T_{1}}\left[e^{-M{\left(\max\left\{z,T_{0}\right\}-z\right)}^{\beta -1}}-e^{-M{\left(T_{1}-z\right)}^{\beta }}\right]l\lambda e^{-l\lambda (z-s)}dz$$

which we solve numerically by quadrature using the *integrate* routine from R [21].

[custom]

## References

[1] Teixeira A.P., Soares C.G., Netto T.A., Estefen S.F. (2008). Reliability of pipelines with corrosion defects. Int. J. Press. Vessel. Pip..

[2] Amaya-Gómez R., Sánchez-Silva M., Bastidas-Arteaga E., Schoefs F., Muñoz F. (2019). Reliability assessments of corroded pipelines based on internal pressure—A review. Eng. Fail. Anal..

[3] Bouledroua O., Zelmati D., Hassani M. (2019). Inspections, statistical and reliability assessment study of corroded pipeline. Eng. Fail. Anal..

[4] Faroz S.A., Pujari N.N., Ghosh S. (2016). Reliability of a corroded RC beam based on Bayesian updating of the corrosion model. Eng. Struct..

[5] Jia S., Akiyama M., Frangopol D.M., Xu Z. (2024). Bayesian inference of the spatial distribution of steel corrosion in reinforced concrete structures using corrosion-induced crack width. Struct. Saf..

[6] Xu H., Gardoni P. (2016). Probabilistic capacity and seismic demand models and fragility estimates for reinforced concrete buildings based on three-dimensional analyses. Eng. Struct..

[7] Chen D.G., Lio Y., Ng H.K.T., Tsai T.R. (2017). Statistical Modeling for Degradation Data.

[8] Hermann S., Ruggeri F. (2017). Modeling wear in cylinder liners. Qual. Reliab. Eng. Int..

[9] Martz H.F., Waller R.A. (1982). Bayesian Reliability Analysis.

[10] Hamada M.S., Wilson A.G., Reese S.C., Martz H.F. (2008). Bayesian Reliability.

[11] Gelman A., Carlin J.B., Stern H.S., Dunson D.B., Vehtari A., Rubin D.B. (2015). Bayesian Data Analysis.

[12] Saaty T.L. (1994). Fundamentals of Decision Making and Priority Theory with the Analytic Hierarchy Process.

[13] Saaty T.L. (1980). The Analytic Hierarchy Process.

[14] Saaty T.L. (1994). Highlights and critical points in the theory and application of the Analytic Hierarchy Process. Eur. J. Oper. Res..

[15] Vaidya O.S., Kumar S. (2006). Analytic hierarchy process: An overview of applications. Eur. J. Oper. Res..

[16] Cagno E., Caron F., Mancini M., Ruggeri F. (2000). Using AHP in determining the prior distributions on gas pipeline failures in a robust Bayesian approach. Reliab. Eng. Syst. Saf..

[17] Kutoyants Y. (2023). Introduction to the Statistics of Poisson Processes and Applications.

[18] Rios Insua D., Ruggeri F., Wiper M.P. (2012). Bayesian Analysis of Stochastic Processes Models.

[19] Winkler R.L. (1967). The assessment of prior distributions in Bayesian analysis. J. Am. Stat. Assoc..

[20] Geyer C.J., Johnson L.T. *MCMC: Markov Chain Monte Carlo*. R Package Version 0.9-8. https://CRAN.R-project.org/package=mcmc.

[21] R Core Team (2013). R: A Language and Environment for Statistical Computing.

